# Fatigue and disturbances of sleep in patients with osteogenesis imperfecta – a cross-sectional questionnaire study

**DOI:** 10.1186/s12891-017-1922-5

**Published:** 2018-01-08

**Authors:** Heidi Arponen, Janna Waltimo-Sirén, Helena Valta, Outi Mäkitie

**Affiliations:** 1 Department of Oral and Maxillofacial Diseases, University of Helsinki, P.O. Box 41, FI-00014 Helsinki, Finland; 2 Department of Oral and Maxillofacial Diseases, Helsinki University Hospital, Helsinki, Finland; 3 Children’s Hospital, University of Helsinki and Helsinki University Hospital, Helsinki, Finland; 4 Folkhälsan Research Center, Helsinki, Finland; 5 Center for Molecular Medicine, Karolinska Institutet, and Department of Clinical Genetics, Karolinska University Hospital, Stockholm, Sweden

**Keywords:** Osteogenesis imperfecta, Fatigue, Sleep disturbances, Quality of life

## Abstract

**Background:**

Persisting fatigue has been reported to be a common complaint by individuals with connective tissue disorders, including Osteogenesis imperfecta (OI). This controlled study evaluated in an adult OI population the subjective experience of fatigue, affecting daily life. Sleep disturbances and chronic pain were examined as hypothesized underlying factors.

**Methods:**

This cross-sectional study analyzed the answers of 56 OI patients and 56 matched healthy controls to a questionnaire, designed to evaluate levels of experienced fatigue and bodily pain, as well as the presence or absence of symptoms related to sleep disturbances or sleep apnea. The relationships between fatigue, pain, and sleep disturbances were evaluated with correlation analysis and regression analysis.

**Results:**

Fatigue was reported by 96%, and daily pain by 87% of the individuals with OI. Notably, the level of fatigue was similarly experienced by patient respondents and controls. In total, 95% of the patients and 77% of the controls reported one to several sleep disturbance symptoms. These symptoms as well as previously diagnosed sleep apnea were statistically significantly more prevalent in the patient group than in the controls (*p* < 0.05). Likewise, the experienced bodily pain was statistically highly significantly more severe among the respondents with OI (*p* < 0.001), and correlated with the reported fatigue.

**Conclusions:**

In comparison with age-matched controls, adults with OI do not differ in experienced fatigue, unlike hypothesized. Therefore, sleep disturbances, which based on the frequency of reported related symptoms and previous sleep apnea diagnoses appear to be common in OI patients, may remain undiagnosed.

**Electronic supplementary material:**

The online version of this article (10.1186/s12891-017-1922-5) contains supplementary material, which is available to authorized users.

## Background

Osteogenesis imperfecta (OI) is a group of genetic disorders of collagen synthesis, characterized by osteoporosis and increased fracture tendency. OI affects approximately one in 10,000 people [1]. The clinical phenotype of OI ranges from mild to severe, the mild type being the most common. The original Sillence classification designated the mild type as type I, the perinatally lethal as type II, the severe, progressively deforming one as type III, and the moderate one as type IV OI. [2, 3]. Despite the subsequent addition of histologically, radiologically, and genetically distinct OI types, autosomal dominant mutations in COL1A1 and COL1A2 still account for approximately 90% of OI cases, and the patients can be classified according to phenotypic severity into the originally defined four types [4, 5]. Particularly in the moderate and severe forms of OI, recurrent long-bone fractures and vertebral compression fractures commonly result in skeletal deformities and scoliosis, leading to short stature and physical disability. Potential extra-skeletal manifestations of OI include cardiovascular complications, pulmonary dysfunction, neurologic problems, hearing loss, dental and occlusal problems, pain, and fatigue. These co-morbidities, or a fear of them, affect in several ways the patients’ quality of life and functional ability [6–9].

The Constitution of World Health Organization (WHO) defines quality of life (QoL) as " individuals’ perception of their position in life in the context of the culture and value systems in which they live and in relation to their goals, expectations, standards and concerns”. QoL is a broad, multidimensional concept that includes subjective evaluations of both positive and negative aspects of life. OI affects not only the QoL of the patient, but also that of a caregiver. [8, 10, 11]. Previous research indicates that QoL of individuals with OI is negatively influenced by reduced function due to tiredness and fatigue [8, 9]. Yet, the prevalence and experience of fatigue in patients with OI remains largely unexplored.

The aim of this cross-sectional study was to examine in an adult OI population the subjective experience of fatigue, affecting the patients’ everyday life, and to evaluate in particular the level of pain and prevalence of self-reported sleep disturbances, in order to assess their role in excessive daytime sleepiness and fatigue. The hypothesis is that patients with OI in comparison to healthy controls report higher levels of both fatigue and pain, and that pain, poor quality of sleep, and fatigue form a vicious circle, impairing the QoL in OI.

## Methods

The study was approved by the Research Ethics Committee, Helsinki University Hospital. We constructed a custom questionnaire to assess the subjective perception of fatigue, bodily pain, and sleep disturbance symptoms. The questionnaire was posted to the unselected 151 individuals, registered as patient members of the Finnish Osteogenesis Imperfecta Association. Inclusion criteria were diagnosis of OI, and age of 16 years or above. Data were unavailable of those Association members who did not return the study questionnaire.

The survey was presented in the following sequence: (1) Collection of general demographic and anthropometric information, and subjective assessments on experienced daily pain and fatigue (Table 1); (2) collection of specific information on symptoms related to fatigue and sleep disturbances (Table 2); and (3) qualitative analysis of subjective reports on sleep related problems. Demographic data included age and gender. Height and weight were enquired as anthropometric information, and were used to calculate the body mass index (BMI). Medical information addressed OI type according to Sillence classification, primary means of mobility, medical diagnosis of sleep apnea confirmed by a polysomnography, and sleep disturbance symptoms. Pain and fatigue were evaluated with a visual analogue scale (VAS) from 0 to 10, where 0 is no pain/fatigue and 10 is the worst possible pain/fatigue a person can imagine. To establish the level of general physical impairment and functional performance, ambulation was assessed as walking without aid, walking with crutches, or wheelchair as a primary mode of mobility. The other variables, presented in Table 2, were dichotomous (yes/no). This study used additional qualitative analysis in order to gain insight into any factor affecting sleep. Qualitative data were collected with one open-ended question:” Do you have other sleep-related problems?”. The same questionnaire, excluding anthropometric information to increase unidentifiability of the participants, was completed by 56 age- and gender-matched volunteer controls without OI.

**Table 1:** Characteristics of the Osteogenesis imperfecta (OI) patient cohort, sub-grouped by OI type, and of the healthy controls

	All	Mild type	Moderate type	Severe type	Unknown type	Controls
Number of participants	56	22	7	10	17	56
Age range in years (mean)	16–75 (47)	19–75 (48)	26–55 (43)	18–52 (33)	16–71 (54)	16–75 (44)
Females/males	41/15	19/3	7/0	5/5	10/7	41/15
Height range in meters (mean)	0.80–1.75 (1.45)	1.47–1.75 (1.6)	0.80–1.65 (1.3)	0.80–1.33 (1.1)	1.10–1.75 (1.5)	NA
Body mass index, mean	29	27	28	36	29	NA
Ambulation (no aid/crutches/wheelchair)	31/5/19	19/2/1	3/0/3	0/0/8	9/3/8	56/0/0
Bodily pain range (mean)	0–10 (4)	0–9 (4)	0–10 (4)	0–5 (2.3)	0–9 (4)	0–9(2)^a^
Fatigue range (mean)	0–10 (5)	2–10 (6)	2–8 (5.8)	1–7 (2.9)	0–8 (5)	0–8 (4)
Diagnosed sleep apnea	8	2	1	2	3	1

*NA* data not requested

^a^the difference, as compared to patient group mean, is statistically highly significant as *p* < 0.001

**Table 2:** Prevalence of self-reported symptoms related to sleep-disturbances and sleep apnea among the respondents in the Osteogenesis imperfecta patient cohort and controls

	Number of patients with symptoms (%) *N* = 56	Number of controls with symptoms (%) *N* = 56	Significance of difference between the study groups (Chi-Square test)
Snoring	32/52 (62)	15/54 (28)	11.1 *p* = 0.001
Pauses of breathing during sleep	13/42 (31)	3/54 (6)	9.9 *p* = 0.002
Restless sleep	43/55 (78)	23/54 (43)	12.9 *p* < 0.001
Grinding of teeth during sleep	22/52 (42)	23/55 (42)	Non-significant
Recurrent nightmares	16/50 (32)	2/55 (4)	13.3 *p* < 0.001
Daytime sleepiness	43/53 (81)	34/56 (61)	5.6 *p* = 0.018
Likelihood of dozing	14/52 (27)	5/56 (9)	6.3 *p* = 0.012
Dysphagia	17/51 (33)	1/55(2)	20.0 *p* < 0.001
Difficulties of concentration	24/50 (48)	6/54 (11)	16.6 *p* < 0.001
Nocturia	15/51 (29)	5/54 (9)	5.8 *p* = 0.016
Restless legs	19/51 (37)	8/54 (15)	10.0 *p* = 0.002
Diagnosed sleep apnea	8/54 (15)	1/55 (2)	5.4 *p* = 0.020

Descriptive data are given as means, ranges, frequencies, and percentages. Height Z-score, reflecting the general severity of the disorder [12], was calculated and used in the statistical analysis. Pearson and Spearman correlation coefficients were computed, according to the distribution of the data, to assess the relationship between the variables in SPSS (IBM Corp. Released 2015. IBM SPSS Statistics for Windows, Version 23.0. Armonk, NY: IBM Corp.) The relationship between sleep disturbance symptoms and fatigue was evaluated by means of logistic regression analysis. Explanatory variables were: diagnosed sleep apnea, snoring, pauses of breathing during sleep, restless sleep, grinding of teeth during sleep, recurrent nightmares, daytime sleepiness, likelihood of dozing, dysphagia, difficulties of concentration, nocturia, and restless legs. Two-sample t-test and chi-square test were used to compare the responses of the patients and controls.

## Results

The study group comprised 56 individuals with OI completing the survey (37% of those who received the questionnaire), and 56 healthy controls. Of the respondents, 39 had a classified OI, and of them 22 had type I (mild), 7 had type IV (moderate), and 10 had type III OI (severe) (Table 1). Altogether 17 reported OI type to be unknown. Of the respondents, 41 were females and 15 males. Their age distribution showed that middle-aged and older individuals were active participants. In total, 20% of the respondents were in the age range of 16–29 years old, 32% were in the age range of 30–49 years, and 48% were aged 50 years or more. Mean age was somewhat lower in those with severe OI (34 years) compared to those with mild OI type (48 years). The majority of the respondents walked unaided (55%). Those with a more severe OI type utilized more often mobility aids. Wheelchair was the primary means of mobility for 19 patients (34%).

Table 1 outlines the notable variation in the reported current bodily pain and fatigue, with an average score of 5 for fatigue and 4 for pain for the patient group, corresponding to a moderate intensity of fatigue and pain. Fatigue and bodily pain were positively correlated [r(55) = 0.29, *p* = 0.034]. No correlation between bodily pain and severity of OI was found, whereas fatigue and severity of OI were negatively correlated [r_s_(38) = −0.34, *p* = 0.037], indicating more reports of severe fatigue among those with a mild OI type. In the youngest patient age-group, the average score was 2 for pain and 4 for fatigue. In the age-group of 30–49 year-olds, the average score was 4 for pain and 5 for fatigue. In the oldest age group, the average scores for pain and fatigue were 5.

Of the 54 participants with OI, who responded to the dichotomous question about the presence or absence of sleep apnea, eight (15%) had diagnosed sleep apnea and used a positive airway pressure ventilator during sleep. Yet of the remaining 46 without diagnosis of sleep apnea, as many as 44 (96%) reported one or more sleep disturbance related symptoms, daytime sleepiness (in 81%) being the most common one (Table 2). BMI and height Z-score showed a negative correlation [r(56) = −0.38, *p* = 0.004], implying that the more severe the OI is, the higher the BMI. No correlation was found between height Z-score and presence of sleep apnea. In contrast, BMI correlated positively with sleep apnea [r_s_(54) = 0.28, *p* = 0.043]. The average scores for fatigue and pain were similar in the eight patients with sleep apnea diagnosis and those without a diagnosis (5 for fatigue and 4 for pain).

Logistic regression analysis showed that in patients with OI, a risk of higher levels of fatigue correlated with daytime sleepiness (OR 3.8; 95%CI: 1.77–6.01; *p* = 0.001) but the risk was not associated with the other sleep disturbance symptoms. Those OI respondents with pauses of breathing during sleep had significantly more frequently diagnosed sleep apnea [r_s_(42) = 0.46, *p* = 0.002], habit of snoring [r_s_(42) = 0.51, p = 0.001], and high likelihood of dozing [r_s_(41) = 0.47, p = 0.002], as can be anticipated. In contrast, pauses of breathing and daytime sleepiness did not show a correlation [r_s_(40) = 0.25, *p* = 0.117].

OI patients were afflicted by statistically significantly more severe bodily pain than the controls (t_102_ = 4.6, *p* < 0.001), whereas the level of experienced fatigue did not differ significantly between the patient and control groups. Among the controls, the average scores for fatigue were similar in all age groups; 3.4 for those aged 29 years or below, 3.8 for those between 30 and 49 years, and 3.7 for those aged 50 years or above. Of the controls, 73% reported symptoms related to sleep disturbances. Statistically significantly more common in the OI patient group than in the controls were snoring, pauses of breathing during sleep, restless sleep, recurrent nightmares, daytime sleepiness, likelihood of dozing, dysphagia, difficulties of concentration, nocturia, and restless legs, whereas grinding of teeth during sleep was equally prevalent (Table 2).

In addition, answers to the open-ended question revealed that eleven respondents with OI suffered from frequent awakenings and four from difficulties in falling asleep. Other reported sleep disturbances among the OI patients were insomnia, nocturnal sweating, pain-induced awakening, numbness of limbs and cranium, depression, anxiety, and awakening by a feeling of falling or choking. One healthy control detailed nocturnal sweating, and another foot spasms as sleep-related disruptive events.

## Discussion

This study found self-reported fatigue of various severity in 96% of individuals with OI. Fatigue was similarly experienced by respondents of all ages. Altogether 76% of the patients also reported symptoms that suggest the presence of a sleep disturbance such as sleep apnea. Daily pain was reported by 87% in the patient cohort. The older patients reported more severe pain than the younger ones. A subjective feeling of fatigue was interestingly an equally current complaint in the control group. Among the control respondents experiencing fatigue, 86% also reported at least one symptom related to sleep disturbances.

Fatigue is considered a more long-term condition than somnolence [13]. It can have multiple physical and mental causes. Fatigue appears to be a common finding in patients with connective tissue disorders when compared to the general population [14]. In patients with Marfan and Ehlers-Danlos syndromes higher levels of fatigue have been shown to be associated with chronic pain [14, 15]. Similarly, in our patient cohort, the severities of fatigue and pain were expectedly positively correlated. To our knowledge, the underlying causes of fatigue, apart from physical fitness, had not previously been studied in OI population.

Presumably low muscle function and level of physical activity contribute to fatigue. Restrictions of physical activity due to recurrent fractures, skeletal deformities and pulmonary dysfunction may lead to decreased functional ability, and furthermore aggravate fatigue in individuals with OI. In patients with mild OI (type I), no pulmonary or cardiac symptoms at rest are normally present. Previous studies, however, show that muscle size, exercise tolerance and muscle strength are significantly reduced in patients with OI [16–20]. On the other hand, training has been shown to improve aerobic capacity and muscle force, and to reduce the level of subjective fatigue in children with mild and moderate OI types [21]. In the light of previous studies, our finding of lower average fatigue score in the individuals with severe OI type, in contrast to those with mild type, was unexpected. It is still unclear how the fatigue and severity of OI relate to each other. One possible explanation for our seemingly contradictory finding could be that those individuals with mild OI type might have higher social participation level than those with severe type. The following hypothetical assumption would be that for instance occupational participation alongside general population demands considerable effort, leading to exhaustion. In addition, importantly, respondents with mild OI were on average 14 years older than those with severe type, which might contribute to their fatigue level - although the reported fatigue among the control group did not correlate with respondents’ age, emphasizing the subjective aspect of fatigability. In patients with OI, we found the severity of fatigue not correlating with the presence of sleep disturbance symptoms other than daytime sleepiness.

Chronic pain is intertwined with insomnia, psychological distress, difficulties in falling asleep, and frequent arousal [22, 23]. Our results agree with these findings. Insomnia, defined as a difficulty of falling asleep or staying asleep, was reported by 25% of the patient respondents in our cohort in the answers to the open-ended question. All of them were also suffering from a generalized pain. Interestingly however, the two patients who declared having anxiety and depression reported low scores for fatigue and pain. The answers obtained from the open-ended question are consistent with the trend of the quantitative data results, indicating a considerable variability and subjectivity of the experience of pain and fatigue.

Individuals with varying severity of OI face several QoL challenges across their lifespan. OI is conjoint with high prevalence of co-morbidities in musculoskeletal, auditory, pulmonary, and endocrine systems, as well as dental problems and physical appearance alterations, all of which are factors that can affect individuals’ QoL [9]. Earlier studies have revealed that adults with OI experience lower physical QoL but similar or higher levels of mental QoL than the general population [24]. The severity of OI has been found to correlate with worsening of QoL. Experiencing pain, isolation, activity limitations and participation limitations have been shown to negatively affect QoL in OI. High scholastic competence has been found in children and adults with OI. The risk of fractures is an important concern faced by both adults and children [11, 24]. This study found several of the previously mentioned QoL risk factors, such as experienced pain and activity limitations in the OI population.

Sleep apnea is a sleep disorder characterized by pauses in breathing or instances of shallow or infrequent breathing during sleep. Sleep apnea is classified as central, obstructive, or mixed. The diagnosis of sleep apnea is based on the conjoint evaluation of clinical symptoms and of the results of a polysomnography as apneic events per hour of sleep [25]. Many of the symptoms of sleep apnea are non-specific and can have other possible causes. Sleep apnea affects roughly 3% of middle-aged men and 2% of women in Finland [26]. In comparison, a self-reported 14% prevalence of sleep apnea in an adult OI population in US has been reported [9]. Similarly, in our cohort, 15% of the respondents had a diagnosed sleep apnea, confirmed by a polysomnography. General factors predisposing to sleep apnea are obesity, snoring, male gender, advanced age, structural abnormalities in the nose and pharynx, nasal obstruction, small or receding lower jaw, functional factors such as small lung capacity, and unstable ventilatory control during sleep [27, 28]. In our OI cohort, BMI exceeded the WHO diagnostic threshold for obesity (30 kg/m^2^) in 33%. The use of BMI-measure can be misleading in individuals with scoliosis and lower extremity deformities that lead to loss of height, since the index is a height-related measure. Location of fat deposition, especially anterolateral to the upper airway, is more important than BMI in regard to risk for obstructive sleep apnea (OSA) [29]. Findings on the association between BMI and excessive daytime sleepiness are inconsistent in the literature when the effect of OSA is not considered [30, 31]. Daytime sleepiness has been associated with depression, nocturia, and metabolic factors more strongly than with sleep-disordered breathing or sleep disruption per se. Individuals with sleep apnea are not always pathologically sleepy [30, 32].

Snoring was reported by 62% of the OI respondents. Snoring is a poor predictor of sleep apnea due to its commonness in the general population. Of men 35–45% and of women 15–28% report habitual snoring. The absence of snoring, however, makes apnea unlikely; only 6% of apnea patients do not snore [29]. Insomnia and restless legs syndrome are each clinically significant in 5–10% of adults in general population, and an average incidence of 23% of respiratory-related leg movements has been documented in a sleep apnea cohort [33–35]. According to this study, insomnia and restless legs are more frequent in OI population than among unaffected peers.

This study presented with several strengths and limitations. We used a mixed-method subjective self-evaluation approach, using a non-validated detailed questionnaire for not to miss any data that might help us to understand the subjective experience of fatigue. The visual analogue scale (VAS) is a simple and frequently used method for assessment of intensity of pain [36]. Similarly, VAS has been shown to be a valid and reliable instrument for the quantitative assessment of fatigue in both healthy subjects and in patients who complain of poor-quality sleep [37]. The possible use of psychoactive drugs affecting sleep was not documented. Without knowledge of the pharmaceutical history of the patients, the association of possible bisphosphonate treatment and experienced bodily pain could not be evaluated. The younger patients and those with severe OI are more likely to have been treated with bisphosphonates that have been shown to be effective in reducing bone pain and fatigue [38, 39]. The study population was limited due to the rarity of OI. Although the disease equally affects both genders, females were more active than males in participating. Possible response bias cannot be evaluated, as the investigators did not have access to the entire target population’s characteristics and medical history. It is likely that those with more symptoms were more likely to participate. However, more than one third of the target population responded and the frequency of symptoms was very high, indicating that the studied symptoms involve a significant proportion of the entire OI population.

## Conclusions

From the outcome of our investigation it is possible to conclude that sleep disturbances are relatively common symptoms in adults with OI, and more prevalent in adults with OI than in the general population. Subjective feeling of fatigue was equally experienced among the healthy controls and in patients with OI. There are several possible explanations for this result. Persistent fatigue that affects day-to-day life was experienced by respondents of all ages. The data gathered show that 76% of those OI patients suffering from fatigue also reported symptoms that suggest the presence of a sleep disturbance such as sleep apnea. However, only 15% of the respondents with OI had a sleep apnea diagnosis. Therefore, further studies of the true prevalence, causes, and characteristics of sleep disturbances in OI population would be of interest.

## Additional file

Additional file 1: Fatigue and disturbances of sleep in patients with Osteogenesis imperfecta –survey questionnaire. (DOCX 102 kb)

## Abbreviations

OI: Osteogenesis imperfecta
OSA: Obstructive sleep apnea
QoL: Quality of life
