# Supplementary material for: Fatigue and disturbances of sleep in patients with osteogenesis imperfecta – a cross-sectional questionnaire study
Source: BMC Musculoskelet Disord. 2018 Jan 8;19:3. doi: 10.1186/s12891-017-1922-5 (PMC5759205; doi:10.1186/s12891-017-1922-5)
Supplement: Additional file 1: — Fatigue and disturbances of sleep in patients with Osteogenesis imperfecta –survey questionnaire. (DOCX 102 kb) [file 12891_2017_1922_MOESM1_ESM.docx]

# **Fatigue and disturbances of sleep in patients with Osteogenesis imperfecta –survey questionnaire**

## Medical background

| Age |  | |
| --- | --- | --- |
| Gender |  | |
| Height |  | |
| Weight |  | |
| OI-type (if known) |  | |
| Primary mode of mobility (walking without aid/walking with cane or crutches/wheelchair) |  | |
|  |  |  |
| **Assess the intensity of following symptoms with a scale from 0 to 10** |  |  |
| Chronic bodily pain | **0---1---2---3---4---5---6---7---8---9---10** | |
| 0= no pain, 10= worst pain possible | | |
| Fatigue /persisting tiredness | **0---1---2---3---4---5---6---7---8---9---10** | |
| 0= no fatigue, 10= severe exhaustion and fatigue | | |
|  | **Yes** | **No** |
| I have been diagnosed with sleep apnea |  |  |
| I would like to take a sleep study |  |  |
| I would like to be contacted by phone/email/mail. | Contact information: |  |

Your are welcome to give any additional information on a separate sheet of paper.

**Tick a box next to each symptom if you experience it on a weekly basis**

|  | Yes | No | Additional information |
| --- | --- | --- | --- |
| Snoring |  |  |  |
| Pauses of breathing during sleep |  |  |  |
| Restless sleep |  |  |  |
| Grinding of teeth during sleep |  |  |  |
| Recurrent nightmares |  |  |  |
| Daytime sleepiness |  |  |  |
| Tendency to doze off |  |  |  |
| Difficulty in swallowing |  |  |  |
| Difficulties of concentration |  |  |  |
| Frequent urination at night |  |  |  |
| Restless legs |  |  |  |
| Do you have other sleep-related problems? |  |  |  |
